# Ideal body image and socioeconomic factors: exploring the perceptions of Kenyan women

**DOI:** 10.1186/s12905-024-03307-5

**Published:** 2024-09-11

**Authors:** Linette Waltsgott, Adekunle Adedeji, Johanna Buchcik

**Affiliations:** 1 Faculty of Life Sciences, Department Health Sciences, Hamburg University of Applied Sciences, Hamburg, Germany; 2 Bremen International Graduate School of Social Sciences (BIGSSS), Constructor University, Bremen, Germany; 3 Department of Medical Psychology, University Medical Center, Hamburg-Eppendorf, Hamburg, Germany

**Keywords:** Ideal Body Image, Obesity, Public perception, Socioeconomic status, Kenya, Tradition

## Abstract

**Background:**

Non-communicable diseases are an increasing threat in sub-Saharan Africa (SSA), and overweight and obesity are affecting people across all socioeconomic groups. Some studies suggest that big body sizes may be perceived as desirable among women in SSA and that high prevalence of obesity and overweight are especially present in low socioeconomic societies. This study explores the role of socioeconomic factors in the perception of the ideal body among Kenyan women and whether perceptions and beliefs about the ideal body should be considered relevant when targeting the prevention of obesity and overweight.

**Method:**

In-depth interviews were conducted with 8 Kenyan women with varying educational backgrounds, aged between 21 and 48, using a qualitative study design. The interviews were conducted in December 2022 and January 2023 in Nairobi, audio-recorded, transcribed and analysed through qualitative content analysis and a coding system using deductive and inductive codes.

**Results:**

The participants reported that conclusions about a person’s health and wealth status are drawn based on different body sizes. Furthermore, traditional views about the ideal body size, societal pressure, as well as the women’s own experience with their body size play a role in the perception of an ideal body.

**Conclusion:**

Small-sized women desire to gain weight as society may view them as weak and sick. Big-sized women aim to reduce weight primarily due to health complications. Nevertheless, traditionally, a big-sized woman is considered strong and wealthy, creating external pressure on women to fulfil this body image—these findings emphasise traditional aspects in designing culturally sensitive prevention and intervention methods to address overweight and obesity.

**Supplementary Information:**

The online version contains supplementary material available at 10.1186/s12905-024-03307-5.

## Background

Overweight and obesity pose massive global health risks, promoting diseases and hampering individual health outcomes [1]. The 2022 World Health Organization (WHO) Report on Health Statistics revealed trends regarding health risks and mortality due to non-communicable diseases (NCDs). Overweight and obesity were identified as significant risk factors for other NCDs, such as cardiovascular diseases (CVD), diabetes, and cancers, among others [1, 2]. This risk is attributed to the metabolic effects on blood pressure, levels of cholesterol and triglycerides, and insulin resistance [3]. Though there has been decline of mortality for NCDs especially in high-income countries, they are still the leading cause of death worldwide [2] Rapid population growth and improved life expectancy raise the total number of deaths to a high level [2]. While the problem of increasing mortality due to NCDs consists all other the world, it is noteworthy that about 77% of all NCD-related mortality is recorded in low- and middle-income countries [4].

In sub-Saharan Africa (SSA), NCDs account for 37% of fatalities [5]. Several studies were conducted to investigate patterns of obesity and overweight in sub-Saharan Africa [6–8]. The results show Kenya as one of the countries with the highest rate of increase in overweight and obesity [8]. In 2010, about one million Kenyan females were living with obesity; the number rose to nearly three million over the last decade [9]. The World Obesity Atlas 2022 further predicts the prevalence of obesity for Kenyan women to be 16.79% in 2030 [9]. Some research has hinted that this trend in Kenya and other SSA countries may partly be attributed to socioeconomic status (SES) and is also more pronounced among women [7, 8]. Noteworthy is that these studies mostly found positive associations between SES, obesity, and overweight [10, 11], meaning that higher SES is associated with a high prevalence of obesity and overweight. These results are further emphasised by several Demographic and Health Surveys (DHS) highlighting socioeconomic characteristics such as economic status and education level as a collate of obesity in low- and middle-income countries [2].

This may be best explained by excess body weight projected association with health and wealth in many parts of sub-Saharan Africa, making excess body weight more desirable [12–15]. Ettarh et al. (2013) find a strong preference for a larger body size among the population of Nairobi slums. The study participants were presented with drawings of body sizes ranging from very thin to very obese. Choosing their ideal body image: more than 30% of the participants chose a desirable body image of being overweight or obese [16]. A similar investigation in Accra, Ghana, allows for the assumption that a bigger body image can be considered more eligible for marriage [14]. In a study conducted among Senegalese women, researchers found that being overweight was the most socially desirable body size [15].

Academic evidence shows that desired body image influences nutritional behaviour [17–20]. Desired thin body image leads to nutritional behaviours such as food restriction to achieve the thin body size [17]. Some studies suggest that this could also hold for achieving larger bodies: A qualitative study by Ndambo et al. (2022) investigated eating behaviour in Malawian women and revealed that women deliberately gain weight to demonstrate good health. Overweight is perceived as an indicator of good health and suggests a successful martial life [21]. Also, Ghanaian women reported that a specific ideal size caused them to attain a certain body size [22]. In 2021, Chigbu et al. investigated the impact of body size perception on obesity and overweight, finding out that most of the population in Nigeria perceive a big body size as desirable. This positive perception impacts the burden of obesity [23].

The results from these above mentioned studies suggest that there may still be broad cultural influences contributing to desirable body size. This conclusion is also supported by studies from Appiah et al. (2016) and Tuoyire et al. (2018) in Ghana, revealing that beyond SES, a specific body image that is seen as desirable according to cultural norms, could also lead to overweight and obesity [13, 22]. Building on this hypothesis, the current study aims to gain an in-depth understanding of Kenyan women and their perception of ideal body image. It further attempts to determine if socioeconomic aspects affect the perception of ideal body image.

This study explores whether women’s perceptions and beliefs about Ideal Body Image and body size should be considered relevant when targeting the prevention of obesity and overweight in Kenya. Through in-depth interviews with Kenyan women, the study investigates whether the perception of body size and socioeconomic status influence ideal body image.

The interviews are structured around the following research questions:

1. What role do socioeconomic factors play in perceiving ideal body image among Kenyan women?
2. Which other factors influence ideal body image?

## Methodology

The research uses an exploratory approach to uncover underlying beliefs and motivations behind preferred body size. This was done using a qualitative research design with a phenomenological approach [24]. This approach allows to get different and detailed perspectives on the topic of interest as described by the participants [24]. The study was conducted via semi-structured and open-ended interviews, allowing for an open and free discussion and the opportunity to retrieve new insights [25].

### Study sample and selection

Convenience, purposeful sampling was used to select female participants with different educational and socioeconomic backgrounds. The study sample comprised 8 Kenyan females aged above 18 and living in Nairobi. The sample size for this approach is sufficient since data saturation and essential elements of meta-themes can be derived after six interviews [26]. Half of the participants have a completed academic degree at a university. The other half have a high school degree or dropped out before graduation. Table 1 overviews the interviewees and their relevant socioeconomics and demographics to the study.

The selection procedure was also intended to represent Kenyan women from different age groups between 18 and 50. Recruitment was conducted at several locations across Nairobi, such as the Great Commissioners Church International in Ngara and the Maasai Market in downtown Nairobi. These are located in downtown Nairobi and were chosen for access to varying age groups and women with sufficient knowledge of the English language.

The interviewees received verbal information about the background of the study, aims, data handling, and data protection. In addition, a written informed consent was signed by all participants. Women with insufficient knowledge of the English language and foreign nationals were excluded from the interviews.

### Data collection

**Fig. 1:**
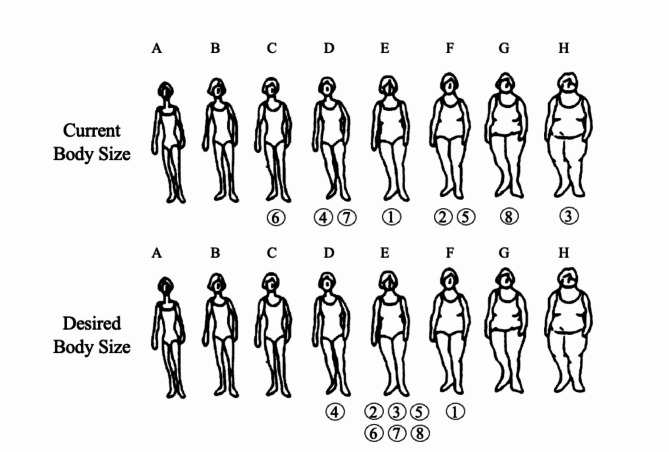
Current Body Size (CBS) & Desired Body Size (DBS) chosen by the women on a set of body silhouettes developed by Stunkard et al. (1983)

Compliance with ethical standards was assessed and approved by the ethical committee of the Competence Center Health of the Hamburg University of Applied Sciences in December 2022. The interview guide developed for this study aims to reveal personal perspectives and beliefs about ideal body image and is built around the main research questions. Using semi-structured questions allows the interviewer to explain the questions to the interviewees in detail and, if necessary, rephrase them in other words for better understanding. Interviews were held in person with only the interviewee and main researcher present to ensure a trustful situation. The interviewees filled out a questionnaire that was developed to provide more information about their socio-cultural background and their body sizes. A set of eight silhouettes was provided (Fig. 1). Body sizes (in the shape of silhouettes) were arranged in increasing order from left to right. They were allocated the letters A-F from left to right for analysis reasons. The interviewees were asked to first choose their current body size. After that, they were asked to choose their desired body size using the same set of silhouettes. The silhouettes were retrieved from Stunkard’s body image figure shapes [27].

The interviews were conducted and audio-recorded between December 2022 and January 2023. The length of the interviews ranged between 9 and 35 min. After conducting the interviews, the recordings were transcribed using the software Otter.ai. The audio recordings were listened to repeatedly for the researchers to familiarise themselves with the content and improve the transcript when the audio quality did not allow the software to transcribe correctly. The process of transcribing followed the rules of the Simplified Transcription System [28].

### Qualitative data analysis

In line with Mayring and Fenzl (2019), this study used structured qualitative content analysis techniques. The main instrument for the analysis is a coding system designed to assign different text passages to categories. A set of deductive codes was developed using the theoretical framework [29] and the research questions. These deductive codes can be summarized in the following categories: *Desired Body Size*,* Actions To Change Towards Desired Body Size*,* Body Size and Wealth*,* Body Size and Health*,* Diet*, and *Factors Influencing Dietary Habits*. The deductive codes were later enhanced with inductive codes based on the data. This two-step approach is well suited for analysing interview data and thus answering theory-driven, open research questions [30]. Data analysis was performed with the software MAXQDA [31] following the coding manual that instructed the coder when to use a specific code and how to differentiate between the codes. After coding 30% of the interviews, the coding manual and deductive codes were reviewed. This yielded new inductive codes (*Body Size and lifestyle*,* Traditional and cultural Beliefs*,* Positive Experience of Small Body Size*,* Negative Experience of Small Body Size*,* Positive Experience of Big Body Size*,* Negative Physical Experience of Big Body Size*,* and Negative Social Experience of Big Body Size*). Therefore, the data allowed to derive parent codes, such as Public Perception and Personal Experience and Diet. After that, the remaining interviews were coded, and the first interviews were re-coded [30]. Finally, possible relationships between different codes and subcodes were further explored.

**Table 1:** Study sample overview, participants aged 21–48

Interviewee	Educational Background	Self-reported BMI	Tribe	Upbringing rural/urban	Occupation
1	Highschool completed	28	Luhya	Urban	Student
2	University Degree	25	Kikuyu	Rural	Lecturer
3	Highschool completed	33	Taita	Rural	Ranger
4	University Degree	20	Kikuyu	Rural	Accountant
5	Highschool completed	28	Kamba	Urban	Salesperson
6	Highschool not completed	17	Luhya	Urban	Salesperson
7	University Degree	21	Kikuyu	Urban	Nutritionist
8	University Degree	38	Kikuyu	Rural	None

## Results

All codes are presented in the Table of Codes below (Table 2). This table further presents the frequency of how many times a given code has been applied and in how many interviews it was mentioned.

**Table 2:** Table of codes

Codename	Code Frequency	Number of Interviewees
Desired Body Size	8	7
Action To Change Towards Desired Body Size	14	8
**Public Perception**		
Body Size & Health	15	5
Body Size & Wealth	32	8
Body Size & Lifestyle	11	6
Traditional & Cultural Beliefs	21	6
**Personal Experience**		
Positive Experience Small Body Size	4	4
Negative Experience Small Body Size	10	5
Positive Experience Big Body Size	10	3
Negative Physical Experience Big Body Size	12	6
Negative Social Experience Big Body Size	10	3
**Diet**		
Eating Behavior & Dietary Habits	16	8
Factors Influencing Dietary Habits	21	8

### Desired body size

As presented in Fig. 1, projecting different body silhouettes that are increasing in size from left to right, three of the 8 participants chose a desired body size (DBS) above their current body size (CBS). None of the interviewees choose the first and second smallest sizes. When asked to choose their DBS, seven out of eight women chose a different size from their current one. Just one woman remained with her current size as the desired size. It was observed that the interviewees choose medium sizes as desired sizes, moving from both smaller and larger sizes towards the middle. Six out of eight interviewees chose E as an ideal size.

Interview extracts further explain this result: “*Not too thin*,* not too fat. At least […] the medium size is okay” - (3)*. “*I am working hard to do some Kgs*,* […] I want to move from here (F) to here (E). But […] I do not want to be skinny.” - (5)* or “*I can’t go down. I can definitely go up. Then I will feel mhh*,* like African (laughs).”- (7)*.

### Action to change towards a certain body size

All participants stated that they take action to achieve their desired body size. In total, 14 coded segments show different actions taken. Six women talked about actions to reduce their current weight, and two women talked about increasing their current weight. Five out of eight participants mentioned that they do more exercise, where a common activity mentioned is walking. Additional measures to work towards a certain body shape lie in the change of the frequency of food intake. Regarding behaviour, two women stated that they adjust their eating patterns by increasing the frequency of their food intake. Being observant about taking all meals and adjusting the daily routine seems a standard measure. Regarding the diet, five women mentioned adjustments in the composition of their meals. They change the diet by cutting down on “unhealthy foods” such as fast foods, sodas, sweets, etc., and including more “healthy” components such as fruits, vegetables, and water and ensuring a balanced diet. Lastly, one woman revealed that she used to be thin in the past and that she used to pray to increase in weight. One woman mentioned using medication to increase her appetite to eat all her meals.

### Public perception

Public perception refers to the public’s collective opinion revealed during the interviews. It includes all codes demonstrating public understanding and attitude towards Ideal Body Image. The concepts that make up the public perception of an Ideal Body Image can be broadly categorised into two categories: socioeconomic and cultural & traditional aspects. Sixty coded segments were retrieved from the interviews comprising the perception of different socioeconomic aspects such as health, wealth and lifestyle and how these link to body size.

Regarding **body size and health**, there is a common understanding among interviewees that small and big body sizes are connected to a person’s health. Almost all women mentioned the negative impacts of large body size, with a focus on diseases such as diabetes, high blood pressure, and other coronary implications that come with excess body weight. Also, three women mentioned physical impairment as they are aware that big body size does not allow people to do their daily activities, and even walking can become a problem. Two women mentioned the occurrence of mental diseases when someone is either one of the extreme body sizes. The questionnaire at the end of the interview reveals that all participants can name at least two risks related to extremely big body sizes. Potential risks linked to a small body size seem less familiar to the interviewees. Given the choice between a thin and a big body, of what is “healthier”, four women choose the thin body size; three, on the other hand, stated a big body size to be “healthier”.

When asked if a **specific body size can be linked to wealth**, five women agreed that a relationship exists between body size and wealth. Four of the participants mentioned that someone who has a big body is considered wealthy in Kenyan society. Big-sized people are doing well financially and are considered more influential and successful. One interviewee shared that the people who drive good cars usually weigh high. One woman explained that doing well is associated with gaining weight. Wealth can allow someone to live a particular lifestyle that leads to gaining weight:

*“Also*,* there is the pressure that […] you are getting some money*,* and you push towards a certain lifestyle which will get you to gain weight. […] If you have wealth […]*,* then you are more sociable; you can just sit and enjoy the food. […] And then also the things that would come with wealth*,* maybe you will be driving*,* […] there is less movement. And also*,* people do not expect you to be walking. So I think wealth and weight […] follow each other” - (7)*.

Wealth is named a contributor to excess body weight. The participants mentioned that a person with more money also has more food.: “I am *getting fat because I have some money. I am feeding myself well.”- (3) *wealthy people can *“eat all the nice things” - (7)*.

Contrarily, the participants also expressed that socioeconomic factors, wealth, and health are related. The participants highlighted that wealth is associated with bigger body size and better health status: “*If you are not wealthy […] you can take even ten years without going for the check-ups. So you cannot know what is going on in your body. “ - (5)*; *“people who are wealthier are better placed” - (1)* and that it is challenging for people from a lower socioeconomic background because medical check-ups are *“still almost not affordable to some people” (4)* even though they are subsidised by the National Health Insurance Fund (NHIF). It is explained that someone’s income will allow them to get their diseases treated.

Six participants linked body size to certain behaviours, habits, and lifestyles. They also highlighted that women are expected to look a certain way due to traditional beliefs and culture. For example: “*Traditionally*,* in my culture*,* if a woman is well built*,* then the husband gets the price. Because the husband has fed the wife. […] Just traditional African*,* that when a woman*,* particularly who is married*,* looks big or has weight*,* then the family is told that they are eating well.“ (2)*. Similarly, conclusions about well-being and financial status are drawn from body size. Two women said that if a wife is brought into the community, they must make sure that the wife is taken care of. One woman shared how the rural community handles this up to date: “*Me as a wife they expect me to look in a certain way. […] There are those people there*,* that community*,* […] when they have the opportunity to feed you*,* they will feed you nicely. […] You know*,* so these two families are really struggling to make sure that this wife they have brought in is doing good. And that is the only way we can show it.” (7)*.

### Personal experience with body size

In total, 46 coded segments are found in all interviews for parent code Personal Experience. Of these, 32 are negative experiences, and 14 are positive experiences. During the interviews, the women were asked to indicate their BMI. Since no measurements were taken, a self-reported BMI was derived by asking the interviewees to estimate their body weight and height. The BMI was categorised according to the World Health Organization’s classification system [32]. When evaluating self-reported BMI according to the classification of the WHO, it was found that three interviewees were classified as having normal weight. Five interviewees are considered overweight and obese (Table 3). Interviewees 1,4,5,6, and 7 spoke about individual experiences with small body sizes. Interviewees 4,5,6 and 7 shared as many positive as negative experiences, while interviewee one only mentioned negative experiences with small body size. “*And somebody will throw in*,* you know*,* comments like you are thin*,* or have you not been eating well*,* or are you stressed*,* and I think such comments get to you sometimes.” - (4)*; “*The whole society is not accepting who I am. So the pressure is always like […] I have to dress something that is going to make me feel fuller.” (7)*.

**Table 3:** Evaluation of self-reported BMI according to WHO classification

BMI	Nutritional status	Self-reported BMI of Interviewee
Below 18.5	Underweight	(6)
18.5–24.9	Normal weight	(4), (7)
25-29.9	Overweight/pre-obesity	(1), (2), (5)
30-34.9	Obesity class I	(3)
35-39.9	Obesity class II	(8)
Above 40	Obesity class III	-

Similarly, five participants shared their experiences with big body sizes. These interviewees, numbers 1,2,3,5 and 8, belong to the WHO classification classes Overweight, Obesity I, and Obesity II (Table 3). Ten coded segments were retrieved for positive experiences with a big body size. Those were shared by interviewees 1,3 and 5. For example: “*If we go collecting firewood*,* me who is heavy*,* I carry so much firewood if I tie up those pieces*,* I carry many compared to those who are weak. […] When we are carrying those sufuria*^1^, *they tell me no*,* you just carry the big one. […] They see that I am physically fit because I’m fat”. (3).* Similarly, negative experiences with big body sizes were shared by all participants. They make up 22 segments. They have negative effects on physical level, as they experience negative effects of being overweight, as well as social levels. “*Yeah*,* because in this day and age*,* most men*,* especially here in Kenya*,* are physically attracted to people with […] big behinds. So you will be walking in town*,* like in a crowded place*,* and somebody will just touch you without consent. Somebody will spank you without consent.” - (8)*

**Fig. 2:**
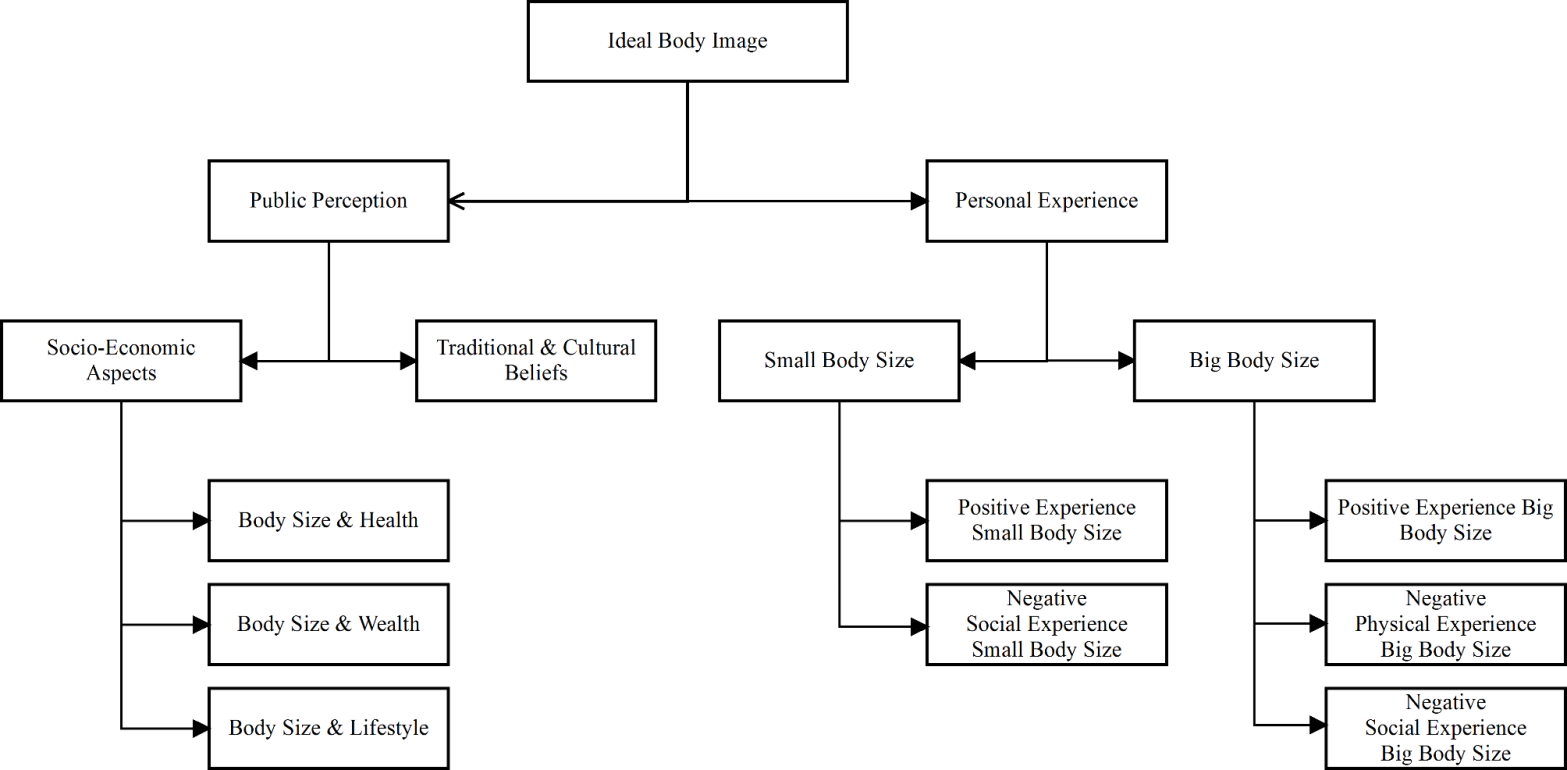
Visualization of two domains of Ideal Body Image, own illustration

### Diet

All participants described their dietary habits and eating patterns. Additionally, they discussed factors that may influence those habits. Most women have a regular structure throughout the week. They eat two to three meals per day and consume mostly the same products and meals. All women follow a diet that mainly comprises staple foods and everything that is locally available. None of the participants mentioned consuming products that cannot be bought in the local markets. Two interviewees mentioned that they do not follow any routine or rules and do not control their eating habits.

The primary factor that influences eating habits for all participants is time. Some are bound to a routine that does not give them a lot of time to cook or eat. Others said that their work environment offers only a limited availability of certain food. Time again was mentioned when it is explained that some dietary habits come from convenience or laziness. Accessibility plays a role as it determines if one can purchase something or if the climate allows them to grow it. Also, availability was often mentioned when they said what is sold on local markets. Economic factors also play a role as costs determine purchasing options and choices. Two women stated that mainly their appetite and cravings determine their choices. Stress and boredom are shared as factors that influence one woman’s eating behaviour. Some mentioned that intolerance or dislikes of certain foods determine their food choices. Also, health aspects were mentioned, and one woman explained that nutrition education influenced her diet.

## Discussion

Results from the in-depth interviews indicate that the perception of the ideal body image is predominantly shaped by two overarching domains: public perception and personal experience, as illustrated in Fig. 2. Within the domain of public perception, participants identified societal standards regarding the ideal female body size as a significant influence. Socioeconomic factors and traditional cultural beliefs influence this perception. Specifically, the socioeconomic aspects project notions of health, wealth, and lifestyle onto body size. Participants noted a prevailing belief within Kenyan society that larger body sizes are associated with affluence and a luxurious lifestyle. Notably, wealth is perceived as contributing to larger body sizes despite assumptions that wealth facilitates better access to healthcare. Conversely, smaller body sizes were often equated with poorer health and inferior socioeconomic status. Additionally, traditional and cultural beliefs differ between urban and rural settings, with urban areas favouring smaller body sizes as more desirable. In comparison, rural communities perceive larger body sizes as indicative of prosperity within marriages and families.

These results could be further related to other findings that suggest exposure to external influences, such as media images, increases body dissatisfaction and may stimulate social comparison to an idealised standard of beauty [33]. Globalization and exposure to Western culture, as argued by Eddy et al. (2007), may be linked to urban preferred body image in our current analysis [34]. More recent findings from Balogun-Mwangi et al. (2023) submit that Kenyan women internalise Eurocentric beauty ideals to some degree [35].

Similar to the results from Mugo (2016), who conducted a study in Kenya’s rural environment, we found that big body size is attributed to being beautiful, healthy and attractive in the rural setting [36]. These results show the significant role the community plays in the execution of traditional customs. The interviews revealed that traditionally, a well-fed wife represents financial prosperity. A wife’s big body size also implies she is doing well in her new community. In summary, these results confirm that traditional and cultural beliefs are essential to addressing body size.

Personal experiences also play a crucial role in shaping perceptions of ideal body image, with participants expressing positive and negative narratives associated with their body size. However, the predominant theme across all body sizes was the prevalence of negative experiences, particularly on the social and physical levels. Smaller-bodied participants reported negative social experiences, while those with larger body sizes experienced both social stigma and physical discomfort. These negative experiences were perceived as exerting significant pressure on individuals to conform to societal norms regarding body size, leading many to aspire a different body size.

These experiences have a significant impact on Ideal Body Image. The women’s exposure to situations where they felt their body size was advantageous led to positive experiences with their body size. On the contrary, situations where it was felt that the body size was of disadvantage caused negative experiences with their body size, which may indicate body dissatisfaction [37]. The negative experiences on a social level stand out as the women report pressure and expectations by their environment. This has also been found in the study of Tuoyire et al. (2018), where the participants were confronted with societal expectations towards their body size [22].

The findings underscore the multifaceted nature of Ideal Body Image formation, influenced by complex interactions between public perception, personal experience, socioeconomic factors, and cultural beliefs. These results contribute to a deeper understanding of the nuanced dynamics shaping body image ideals within the Kenyan context, highlighting the need for interventions that address both external societal pressures and individual experiences to promote healthier and more inclusive body image standards.

### The role of socioeconomic factors in the perception of body size among Kenyan women

The results highlighted that health, wealth, and education are pivotal factors influencing the perception of the ideal body image. Participants expressed concerns that individuals with smaller body sizes might be perceived as undernourished, particularly in a context marked by high rates of poverty and food insecurity, where such perceptions may seem justified [38]. Conversely, there is a widespread awareness among women that larger body sizes carry more significant health risks, including cardiovascular diseases (CVDs) and diabetes. This perception aligns with previous studies wherein women regarded larger body sizes as less healthy [14, 15].

A notable finding from the interviews is the strong association between wealth and body size. Larger body sizes are often construed as indicators of status and financial power. This corroborates findings from earlier research suggesting that body size can be linked to an individual’s socioeconomic status. Additionally, participants noted that wealth facilitates access to a diet rich in unhealthy, high-priced fast food, which contributes to weight gain. The lifestyle associated with wealth, characterised by reduced physical activity and consumption of processed foods and fast food, further reinforces this connection between wealth and body size. This observation resonates with the findings of Mbochi et al. (2012), who found a positive correlation between increased expenditure and higher body mass index (BMI) [39].

The interviews highlight the intricate interplay between health, wealth, and education in shaping body image perceptions. While concerns about the health implications of larger body sizes are prevalent, the association between wealth and body size underscores the socioeconomic factors influencing body image ideals.

### Limitations and strengths of the study

While the study provided valuable insights, it also faced several limitations to consider when interpreting the findings. Firstly, the study’s findings cannot be generalised due to the subjective nature of participant opinions and perceptions. Furthermore, analysis regarding varying body sizes, educational backgrounds, ethnicity and affiliation with tribes was limited due to the sample selection process. Additionally, assumptions about overweight distribution or patterns could not be confirmed, further limiting the scope of the study. Language criteria for inclusion may have excluded some potential participants, introducing cultural barriers. Lastly, personal bias, including social desirability bias, may have influenced participant responses, impacting the reliability of the findings.

Despite these limitations, the study also had several strengths that contributed to its value. It provided valuable insights into the personal views of females in Kenyan society, shedding light on cultural aspects and traditional beliefs influencing women’s self-perception and behaviour. The exploratory approach adopted in the study was one of the first to cover the topic among Nairobi’s female population, paving the way for further research in this area. Moreover, the study highlighted the importance of considering cultural sensitivities in policymaking for disease prevention, emphasising the need for tailored interventions for the mentioned group.

While the study faced certain limitations, its strengths contribute to our understanding of the complex dynamics surrounding body image perceptions and cultural influences among women in Nairobi.

### Recommendations for further research

The results obtained in this study should be supplemented by further research. Socioeconomic factors like wealth, health and lifestyle are associated with the Ideal Body Image and the obtainment thereof by Kenyan women. Further data collection and statistical analysis could shed light on the nature of the relationship and possible causality between the concepts. Shedding some light on the influence of culture and tradition, the findings are intriguing to further investigate the impact of traditional beliefs, as well as the seemingly contradicting influence of globalization on the Ideal Body Image of the younger and urban generation. Is it just beliefes or are there other traditional or local customs that influence eating patterns and behavior? In the analysis of the results, it becomes apparent that there is a difference in the perception of Ideal Body Image and how it is perceived in the communal eye in an urban versus rural setting. Location can play a role, such as the local food environment. The interviewees for this study were all recruited in the city of Nairobi. A next step should be to further investigate rural perceptions and beliefs, as previously done by Mugo et al. (2016) [36], with a new focus on Desired Body Size and its underlying factors.

### Conclusions and practical recommendations

This study concludes that the Ideal Body Image of Kenyan women is significantly influenced by both public perception and personal experiences with body size, which are intricately linked to socioeconomic factors. Larger body size is often associated with status and influence, while societal pressure, including traditional beliefs, can influence desires to gain or lose weight. However, the desire for a medium-sized body is prevalent among big sized women, driven partly by health considerations.

Further research is recommended to investigate the relationship between socioeconomic factors, ideal body image, and health outcomes. Specifically, understanding the causal relationship between wealth, body size, and health complications is crucial. Additionally, exploring the impact of culture and globalisation on body image perceptions among different demographic groups, particularly in urban versus rural settings, is essential.

Moreover, there is a need to investigate health-seeking behaviours among Kenyan women, particularly considering the reported low health-seeking behaviour despite concerns about health implications related to body size. Identifying and targeting populations who can afford healthcare but do not actively seek it is essential for improving healthcare accessibility and informing better policymaking for disease prevention.

## Electronic supplementary material

Below is the link to the electronic supplementary material.

Supplementary Material 1

## Data Availability

The data that support the findings of this study are available on request from the corresponding author, Johanna Buchcik. The data are not publicly available due to information that could compromise the privacy of the study participants.

## Footnotes

1 A sufuria is a cooking pot made out of aluminium.

## Abbreviations

BMI: Body Mass Index
CVD: Cardiovascular diseases
CBS: Current Body Size
DHS: Demographic and Health Surveys
DBS: Desired Body Size
NCD: Non-communicable disease
NHIF: National Health Insurance Fund
SSA: Sub-Saharan Africa
SES: socioeconomic status
WHO: World Health Organization
