# Supplementary material for: Ideal body image and socioeconomic factors: exploring the perceptions of Kenyan women
Source: BMC Womens Health. 2024 Sep 11;24:501. doi: 10.1186/s12905-024-03307-5 (PMC11389067; doi:10.1186/s12905-024-03307-5)
Supplement: Supplementary file 1 — Supplementary Material 1 [file 12905_2024_3307_MOESM1_ESM.docx]

# Interview Guide

**„** **Ideal Body Image and Socio-Economic Status:**

**Exploring perceptions of Kenyan women in a qualitative study“**

Behavioural Patterns:

1. How would you describe your diet and eating habits?
   1. What do you usually eat in a week?
2. What are the main factors influencing these dietary habits?

Perception of Body Image:

1. How would you describe your body size currently?
2. How would you describe your ideal body size?
3. What influences your desired body size/body image?
4. Is there something you do to achieve the body image that you desire?
5. Are there certain attributes that you expect of a person with a big body or thin body?
6. From your perspective, how are body weight and wealth related?
7. How do you think wealth and health are related?
8. Where and when have you felt that your body weight has brought you benefits/advantages or barriers/disadvantages?

# Questionnaire

After the interview *(please fill in the form)*

---------------------------------------------------------------------------------------------

| Age: |  |
| --- | --- |
| Educational background: |  |
| Occupation: |  |
| Cultural upbringing / tribe: |  |
| Grown up in urban or rural setting: |  |
| Self-reported BMI: |  |

Nutrition Education:

1. On a scale from 1-10 *(1= lowest, 10 = highest)* how much do you know about nutrition and health?
2. What is more healthy, thin or big body?
3. What do you think are the health risks related to a thin body? *(please name two aspects)*
4. What do you think are the health risks related to a big body? *(please name two aspects)*

Do you have any questions or anything to add?

# Body Silhouettes

1. How would you describe your body size currently?


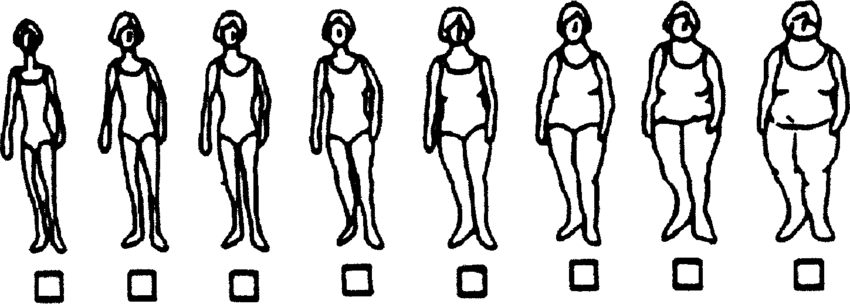


1. How would you describe your Desired Body Size?


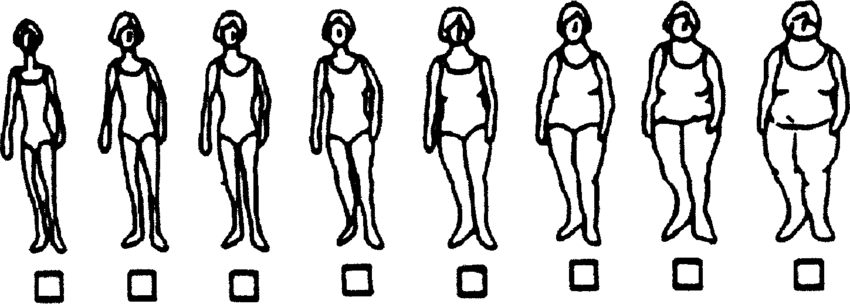


(Stunkard et al., 1983)
